# The Tibial Plateau Map: Fracture Line Morphology of Intra-Articular Proximal Tibial Fractures

**DOI:** 10.1155/2021/9920189

**Published:** 2021-08-24

**Authors:** Maximilian Kerschbaum, Morgane Tyczka, Lisa Klute, Marie Theres Heller, Matthias Koch, Daniel Popp, Siegmund Lang, Volker Alt, Michael Worlicek

**Affiliations:** Department of Trauma Surgery, University Medical Centre Regensburg, Regensburg, Germany

## Abstract

The purpose of this study was to characterize the patterns of a large series of tibial plateau fractures with the use of fracture mapping, with regard to different fracture types using the OTA/AO and Schatzker classification. Patients with intra-articular fractures of the tibial plateau were evaluated, using the OTA/AO and Schatzker classification on CT scans. For fracture mapping, the axial slice that completely displayed the tibial joint plane was first identified, then matched to a template congruently, and the fracture lines were identified and reproduced. In addition to epidemiological data (age and gender), the trauma mechanism (high-energy, low-energy, and pathological fracture) was recorded. In total, 271 patients with 278 intra-articular fractures of the tibial head were analyzed, including seven patients with both sides affected. The mean age was 49.1 years (men 46.3 years, women 53.5 years). The majority of fractures was caused by high-energy trauma. No significant difference could be shown with respect to trauma mechanism and resulting fracture type in terms of OTA/AO (*p* = 0.352) or Schatzker classification (*p* = 0.884). A significant difference could be found with respect to gender and resulting fracture type in terms of OTA/AO (*p* = 0.031). 170 (61.2%) were OTA/AO type B fractures, and 108 (38.8%) were type C fractures. Using the Schatzker classification, we found 53 type I (19.1%), 60 type II (21.6%), 27 type III (9.7%), 32 type IV (11.5%), 16 type V (5.8%), and 90 type VI (32.4%) fractures. The main affection was found in the lateral and intermedial column of the tibial plateaus, concerning both OTA/AO and Schatzker classification. The variability of intra-articular tibial head fractures is very high. In consequence, an individual analysis of fracture patterns and therapy planning by using CT scans is crucial.

## 1. Introduction

Intra-articular fractures of the tibial plateau are common and severe injuries after high impact trauma not only in young patients but also in elderly patients with preexisting osteoporosis [1, 2]. The characteristics of these fractures range from simple split fractures up to complex, multifragmentary fracture morphologies [3–5].

The anatomical reconstruction of these fractures is challenging, but crucial to prevent posttraumatic osteoarthritis [1, 6, 7]. The Schatzker and OTA/AO classification systems are often used to assess these fractures. Although these classifications can be very helpful to gain a first impression of fracture morphology, they have the disadvantage to be based on anteroposterior radiographs, without considering the sagittal and axial plane [8].

To get further information about the articular fracture component and for planning the surgical approach as well as tactics for open reduction and internal fixation, CT scans are indispensable [9].

Cole et al. have described for the first time a novel technique to visualize intra-articular fracture morphology in pilon tibial fractures using CT data sets. They drew a map of fracture zones and comminution zones for each fracture in the axial plane and found a consistent fracture pattern for the majority of AO 43 C3 pilon fractures [10]. This “fracture mapping” has now already been used for distal radial fractures and vertebral body fractures [11, 12]. These studies also showed recurrent fracture patterns and characteristics, which improve the understanding of these fractures and are useful for preoperative planning.

The purpose of this study was to characterize the patterns of a large series of tibial plateau fractures with the use of fracture mapping, with regard to different fracture types using the OTA/AO and Schatzker classification.

## 2. Materials and Methods

**Figure 1:**
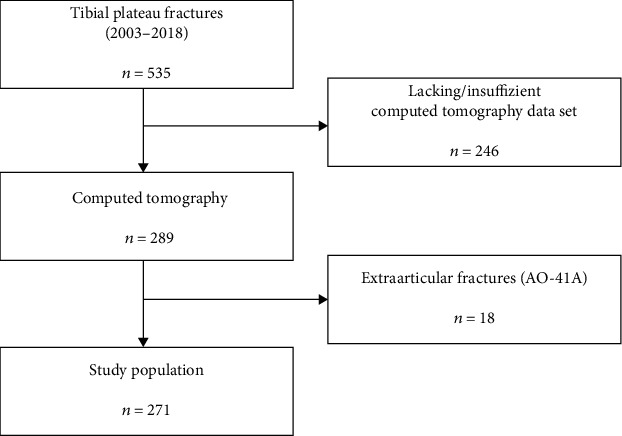
Flowchart of case in- and exclusion. The study population consists only of AO-41B&C fractures.

This investigation was approved by the local Ethics Committee of the University of Regensburg, Germany (No. 20-2059-104). Patients with tibial plateau fractures, diagnosed in our trauma department between 2003 and 2018, were identified. Patients with computed tomography (CT) scans of the affected knee were included. Patients with missing computed tomography data sets or with low image quality were excluded. All fractures were categorized according to the OTA/AO and Schatzker classification systems using the available 3D CT scans. Patients with extra-articular fractures (AO-41A) were excluded in order to generate a study population consisting of intra-articular proximal tibial fractures (AO-41B&C; Schatzker 1-6; Figure 1). In addition to epidemiological data (age and gender), the trauma mechanism (high-energy, low-energy, and pathological fracture) was recorded.

### 2.1. Radiological Evaluation

The radiological evaluation was carried out on the basis of CT scans of the affected knee. Next to axial slices, coronal reconstructions were used for fracture classification (OTA/AO and Schatzker classification). All measurements were performed digitally, using the software package OsiriX MD (Pixmeo, Bernex, Switzerland).

### 2.2. Fracture Mapping

**Figure 2:**
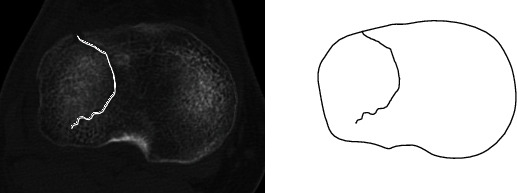
Fracture mapping of a tibial plateau fracture (AO 41-B). Identification of the joint plane in axial slices. Visualization of the identified intra-articular fracture line.

For fracture mapping, the axial slice that completely displays the tibial joint plane was first identified. This axial sectional plane was then transferred to PowerPoint (MSO, 2016). In order to be able to compare the different maps of the tibial plateaus, all slices were brought to the same size and were matched to a template congruently (Figure 2). Left tibial plateaus were virtually mirrored, to have only right tibial plateaus. The fragments were virtually reduced. The fracture lines were then identified and reproduced in detail using PowerPoint (MSO, 2016) (Figure 2).

### 2.3. Data Analysis

The obtained fracture maps were merged according to the OTA/AO and Schatzker classification systems in order to identify regularities of the corresponding fracture entities through the virtual overlay image. Statistical analysis was carried out using SPSS software package version 25 (SPSS Inc., Chicago, Illinois). The chi-square independence test was performed to compare categorical variables. *p* values < 0.05 were considered significant. Cramérs Phi was used to evaluate the effect strength of the differences (low: 0.1 ≤ *w* < 0.3; medium: 0.3 ≤ *w* < 0.5; strong: *w* < 0.5).

## 3. Results

In total, 271 patients with 278 intra-articular fractures of the tibial head were analyzed, including seven patients with both sides affected. The mean age was 49.1 years (men 46.3 years, women 53.5 years). 208 fractures (74.8%) were caused by high-energy trauma, 65 (23.4%) by low-energy trauma, and one was a pathological fracture caused by metastasis (0.4%) (Tables 1 and 2). In 4 patients, the trauma mechanism was not documented (1.4%). No significant difference could be shown with respect to trauma mechanism and resulting fracture type in terms of OTA/AO (*p* = 0.352) or Schatzker classification (*p* = 0.884).

170 (61.2%) were OTA/AO type B fractures, and 108 (38.8%) were type C fractures (Table 1). Using the Schatzker classification, we found 53 type I (19.1%), 60 type II (21.6%), 27 type III (9.7%), 32 type IV (11.5%), 16 type V (5.8%), and 90 type VI (32.4%) fractures (Table 2). The distribution of fracture types according to gender is displayed in Table 3. Significant differences in fracture type between men and women were observed (*p* = 0.031), even with small effect strength ($\text{Cram}\overset{´}{\text{e}}\text{rs Phi}=0.210$).

**Figure 3:**
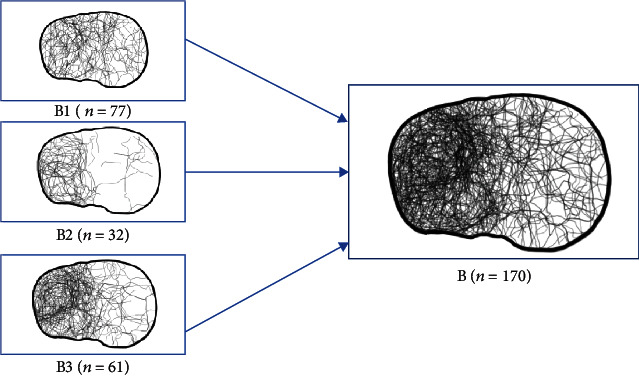
Tibial plateau map of AO-41B fractures.

Concerning the OTA/AO classification, the most common fractures were type B (*n* = 170), with a main affection of the anterolateral, posterolateral, anterolateral central, and posterolateral central segments following the 10-segment classification of Krause et al. [13] (Figure 3).

**Figure 4:**
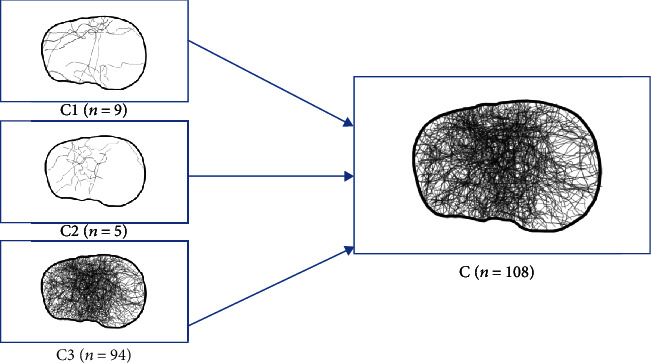
Tibial plateau map of AO-41C fractures.

108 fractures were classified as OTA/AO type C fractures, with a majority of type C3 fractures (*n* = 94; 87.0%) and a main affection of the anterocentral and posterocentral segments following the 10-segment classification of Krause et al. (Figure 4).

**Figure 5:**
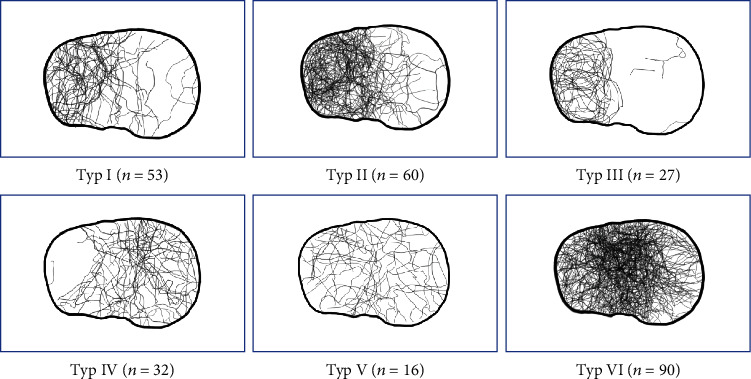
Tibial plateau map of Schatzker 1-6 fractures.

Using the Schatzker classification, we found mostly type VI (*n* = 90; 32.4%), type II (*n* = 60; 21.6%), and type I (*n* = 53; 19.1%) fractures, with also a main affection of anterolateral, posterolateral, anterolateral central, posterolateral central, anterocentral, and posterocentral segments (Figure 5).

## 4. Discussion

The understanding of fracture morphology is crucial for preoperative planning, reduction and fixation of tibial head fractures. In the present study, we analyzed axial CT scans of tibial plateau fractures to investigate the distribution and frequency of fracture lines in a large number of patients. Although there is a two-dimensional assessment of these fractures on anterior-posterior radiographs, using the OTA/AO or Schatzker classification is useful to get a first impression of the fracture severity and morphology [14, 15]; surgery without preoperative CT scans seems unthinkable [2, 4, 16–19]. This is confirmed by our results. Indeed, the fractures could be classified by using these common systems, the fracture mapping showed, and there is a high variability in fracture morphology and distribution of the fracture lines, even within each fracture subgroup.

Several authors have described different classifications and concepts for the assessment of tibial head fractures using axial CT scans and dividing the tibial plateau into different columns or segments [9, 13, 20]. In our daily practice, we commonly use the 10-segment classification developed by Krause et al., as it shows a high reliability in preoperative planning [13].

In accordance with previous studies, we found that mostly the lateral and central area of the tibial plateau seems to be affected, regardless of whether the fractures were classified by the OTA/AO or Schatzker classification system [21, 22]. We also could confirm that the posterior segments are commonly involved in all fracture types, which is an essential information for preoperative planning, choice of surgical approach, and fracture reduction [23].

Interestingly, the trauma mechanism does not play a bigger role for the resulting fracture type. In both groups, we found a majority for OTA/AO type B1 and C3 fractures, respectively, Schatzker I and VI, with especially affected lateral and central segments, but also fracture lines into the medial plateau. This is in accordance with the findings of Krause et al. and Elsoe et al., who found that low-energy trauma resulted in type B fractures as well as in type C fractures [13, 24].

Interestingly, we found a significant difference of fracture types using OTA/AO classification with respect to gender. Type AO-C3 fractures seem to be more common in male patients, whereas the majority of female patients suffer from AO-B fractures. This is in accordance with Krause et al. but needs further investigation [13].

This study has some limitations. Firstly, patients with insufficient or missing CT data sets were excluded. Secondly, the mapping technique can only show the distribution of the fracture lines on the surface of the tibial plateau, so fracture lines descending into distal parts of the tibial head may have been underrated. Thirdly, due to the retrospective character of this study, we did not consider the exact injury mechanism, which led to the fracture, as described by Zhang et al. [25]. Additionally, the fracture map of the tibial plateau shown here cannot display the impression zones of the tibial head.

In conclusion, we showed that the variability of intra-articular tibial head fractures is very high. Although fracture lines occur more frequently in the lateral and intermedial column and the first assessment of the fractures using OTA/AO and Schatzker classification is a helpful tool, an individual analysis of fracture patterns and therapy planning by using CT scans is crucial.

## Figures and Tables

**Table 1:** Presentation of fracture types according to the OTA/AO classification system in relation to the trauma mechanism.

		OTA/AO classification (number of fractures)					
		B1	B2	B3	C1	C2	C3
Trauma mechanism	High energy (#208; 74, 82%)	58	21	47	5	3	74
	Low energy (#65; 23, 38%)	19	10	13	4	2	17
	Pathological (#1; 0, 36%)	—	—	—	—	—	1

**Table 2:** Presentation of fracture types according to the Schatzker classification system in relation to the trauma mechanism.

		Schatzker classification					
		I	II	III	IV	V	VI
Trauma mechanism	High energy (#208; 74, 82%)	40	47	18	22	13	68
	Low energy (#65; 23, 38%)	13	13	8	9	3	19
	Pathological (#1; 0, 36%)	—	—	—	—	—	1

**Table 3:** Presentation of fracture types (OTA/AO classification system) in relation to gender.

		Sex	
		Male	Female
OTA\AO classification	Number of fractures	170 (61.2%)	108 (38.9%)
	B1	47 (27.7%)	30 (27.8%)
	B2	11 (6.5%)	21 (19.4%)
	B3	39 (22.9%)	22 (20,4%)
	C1	7 (4.1%)	2 (1.9%)
	C2	3 (1.8%)	2 (1.9%)
	C3	63 (37.1%)	31 (28.7%)

## Data Availability

The data used to support the findings of this study are included within the article.
